# Neighbourhood Concentration and Representation of Non-European Migrants: New Results from Norway

**DOI:** 10.1007/s10680-019-09522-3

**Published:** 2019-03-12

**Authors:** Adrian F. Rogne, Eva K. Andersson, Bo Malmberg, Torkild H. Lyngstad

**Affiliations:** 1 Department of Sociology and Human Geography, University of Oslo, Oslo, Norway; 2 Department of Human Geography, Stockholm University, Stockholm, Sweden

**Keywords:** Segregation, Comparison, Non-European immigrants, Concentration, Representation, Belgium, Denmark, The Netherlands, Sweden, Norway

## Abstract

In a previous study, Andersson et al. (A comparative study of segregation patterns in Belgium, Denmark, the Netherlands and Sweden: neighbourhood concentration and representation of non-European migrants. Eur J Popul 34:1–25, 2018) compared the patterns of residential segregation between non-European immigrants and the rest of the population in four European countries, using the *k*-nearest neighbours approach to compute comparable measures of segregation. This approach relies on detailed geo-coded data and can be used to assess segregation levels at different neighbourhood scales. This paper updates these findings with results from Norway. Using similar data and methods, we document both similarities and striking differences between the segregation patterns in Norway and Belgium, Denmark, the Netherlands and Sweden. While the segregation patterns in Norway at larger scales are roughly comparable to those found in Denmark, but with higher concentrations of non-European immigrants in the most immigrant-dense large-scale neighbourhoods, the micro-level segregation is much lower in Norway than in the other countries. While an important finding by Andersson et al. (2018) was that segregation levels at the micro-scale of 200 nearest neighbours fell within a narrow band, with a dissimilarity index between 0.475 and 0.512 in the four countries under study, segregation levels at this scale are clearly lower in Norway, with a dissimilarity index of 0.429. We discuss possible explanations for these patterns.

**Electronic supplementary material:**

The online version of this article (10.1007/s10680-019-09522-3) contains supplementary material, which is available to authorized users.

## Introduction

In a special issue of the European Journal of Population, Andersson et al. (2018) presented a comparative study of segregation patterns of non-European migrants in Belgium, Denmark, the Netherlands and Sweden. In all countries, individual-level, geo-coded, register data were used to compute segregation measures based on the *k*-nearest neighbours approach. These individualized scalable neighbourhoods provide comparable neighbourhood definitions and allow for an analysis of segregation patterns at different scales. One important finding of this study was that small-scale segregation patterns, based on neighbourhoods encompassing the 200 nearest neighbours, were remarkably similar across the different countries, with dissimilarity indices (DI) in the 47.5–51.2% range. At larger scales, differences were much more marked. For neighbourhoods encompassing the nearest 51,200 neighbours, the DI in Belgium was 40.6% compared to only 25.3% in Denmark. These findings point to the relevance of scale and suggest that factors influencing segregation at the local level can be different from those that influence segregation at larger scales. A tentative interpretation of these patterns was that similarities in small-scale segregation patterns might be related to the influence of ethnic preferences, whereas differences in housing policies, housing market structure and settlement policies are candidate explanations for a more pronounced large-scale segregation in Belgium.

This research note updates the results from this earlier study by adding results from Norway. In terms of its welfare state structure, Norway is similar to Denmark and Sweden, but differs from these countries in important areas such as settlement policies for refugees and asylum seekers, settlement patterns, de-centralization policies, geography and housing market structure. Thus, extending the analysis of segregation patterns to include Norway will make it possible to evaluate, first, if small-scale segregation in Norway falls within the narrow interval identified for Belgium, Denmark, the Netherlands, and Sweden. And, second, if Norway, with its particular combination of policies, has lower segregation levels and a more even representation of non-European immigrants.

## Background

According to Musterd and Ostendorf (1998), ethnic segregation levels have historically been lower in Europe than in the USA. However, studies of ethnic segregation in Europe have shown large differences between countries. Regarding the countries considered in this research note, Musterd (2005) found relatively low segregation levels in Oslo. Higher levels were found in Belgian cities and for Iranians in Stockholm. Segregation levels for different groups in different Dutch cities varied widely. Musterd and Van Kempen (2009) show relatively high segregation levels for some groups in Dutch and Belgian cities, compared to two Swedish cities. Arbaci (2007) emphasizes the role of welfare regimes in producing patterns of ethnic segregation. Comparing Nordic capitals with similar welfare arrangements, Skifter Andersen et al. (2016) found the highest segregation levels for non-European migrants in Stockholm and the lowest in Helsinki, with Oslo and Copenhagen in between. They emphasize the role of housing markets and housing policies.

Norway is a Nordic welfare state, similar to Denmark and Sweden with regard to policies in areas such as health care, social services, and education, but there are marked differences in some areas that are salient to residential segregation. Immigration has historically been higher in Sweden than in Denmark and Norway. Norway is generally considered to have taken an intermediate position in immigration policy, more restrictive than Sweden and less restrictive than Denmark (Brochmann 2017). Norway’s settlement policies for refugees and asylum seekers have also differed substantively from those of Sweden, but resemble those of Denmark. While Swedish policies emphasize voluntary settlement, asylum seekers and refugees in Norway are for the most part settled in municipalities through a system of agreements between the municipalities and the central authorities (Brochmann 2017; Directorate of Integration and Diversity 2010; Ministry of Justice and Public Security 2016). While they may move freely after this initial settlement, many choose to move to the Oslo region or other major cities (Stambøl 2013). This policy leads us to expect a more dispersed non-European immigrant population in Norway than in Sweden. Further, Norway and Sweden are both larger in geographical terms and have a lower population density than the other countries. The total land area of mainland Norway is approximately eight times that of Denmark and the Netherlands, and ten times that of Belgium, while Sweden is 1.4 times the size of Norway (CIA 2016). While the population density in central areas of Norway and Sweden is high, large areas are sparsely populated or uninhabited. Also, settlement is less centralized in Norway than in Sweden, as a smaller proportion of the Norwegian population resides in urban areas (The World Bank 2018). De-centralization has historically been an important political goal in Norway. Finally, housing policies and the housing market structures in Norway are quite different from those of Sweden, Denmark, the Netherlands and Belgium. In Norway, 77% of households own their dwelling, and public housing only comprises 4% of the housing stock (Statistics Norway na. a, na. b, na. c).

## Data and Methods

The data used here are based on population register data for the entire Norwegian population registered as resident on 1 January 2011, provided by Statistics Norway. The results are compared to corresponding figures from Sweden, Denmark, the Netherlands and Belgium, as described in Andersson et al. (2018). To facilitate comparative analyses, we have aimed to make the data as similar as possible across countries. The process of harmonizing the national data sets is documented in Nielsen et al. (2017) and was the aim of the ResSegr project.^1^

The *k*-nearest neighbours approach to measuring segregation is well suited for comparative analyses, as it provides a comparable definition of a neighbourhood; the *k*-nearest neighbours of each individual. This partially circumvents the Modifiable Areal Unit Problem (Hennerdal and Nielsen 2017) by allowing for a comparison of residential patterns that do not rely on administrative borders. Further, the neighbourhoods are scalable, allowing us to study segregation at both the macro-level (*k* = 51,200), the micro-level (*k* = 200) and at intermediate levels. However, a drawback of this method is that the geographical size of each neighbourhood is determined by the local population density. Thus, the geographical area that is considered a “neighbourhood” is highly variable and may become very large at high *k* values, particularly in less densely populated areas in Norway and Sweden.

The Norwegian data are based on a 100 × 100 m grid covering the entire country, excluding unincorporated areas. We first calculate the total number of individuals and the number of non-European immigrants in each populated grid cell. Non-European immigrants are defined as people born in a non-EU28/EFTA country to two foreign-born parents. Using the specialized software Equipop (Östh 2014), we calculate the proportion of non-European immigrants among the *k*-nearest neighbours of each grid cell, producing a data set consisting of the composition of the egocentric neighbourhoods of each grid cell at different scale levels. The scale levels used here are *k* = 200, *k* = 1600, *k* = 12,800 and *k* = 51,200. In the analyses, these values are weighted by the population count of each grid cell. For *k* = 51,200, a grid of 400 × 400 m cells was used in order to circumvent a technical problem. Table 1 provides descriptive statistics for the grids.

**Table 1:** The gridded population, descriptive statistics, 2011. *Source*: Andersson et al. (2018). Authors’ calculations based on register data from Statistics Belgium, Statistics Denmark, Statistics Netherlands, Statistics Sweden and Statistics Norway. The individual-level data were aggregated to a geographical grid of 100 by 100 m (in Denmark, the Netherlands, Belgium and Norway) and, for Sweden, to a geographical grid of 250 by 250 m (in densely populated areas, due to data restrictions), or 1000 by 1000 m (in sparsely populated areas)

	Number of populated grid squares	Median population	Maximum population	Median number of non-European migrants	Maximum number of non-European migrants	Total population
Belgium	608,850	9	1753	0	516	11,000,638
Denmark	421,365	5	1129	0	275	5,566,100
Netherlands	559,504	11	1105	0	771	16,727,659
Sweden	202,067	157	4114	7	1345	9,466,727
Norway (100 m grid)	456,528	5	573	0	266	4,906,695

As mentioned above, the *k*-nearest neighbours approach produces neighbourhoods that are comparable in terms of population size, but highly variable in geographical size. This is clearly shown in Table 2, which summarizes the geographical size of neighbourhoods at *k* = 200 and *k* = 51,200. Norwegian neighbourhoods at the micro-level of *k* = 200 are roughly similar to those found in the other countries up to the 50th percentile. However, the area covered by many Norwegian neighbourhoods at this scale level is much larger than the areas of neighbourhoods in Belgium, Denmark and the Netherlands. In Norway, 10% of the population live in places where we have to draw a circle with a radius of approximately 1.5 km or more in order to encompass their 200 nearest neighbours. At the macro-level of *k* = 51,200, the Norwegian neighbourhoods are much larger in size than those of Belgium, Denmark and the Netherlands across most of the distribution, but they are comparable in size to Swedish neighbourhoods.

**Table 2:** Size of individualized neighbourhoods in Belgium, Denmark, the Netherlands, Sweden and Norway, radius in metres (percentiles based on population count), 2011. *Source*: Andersson et al. (2018). Authors’ calculations based on register data from Statistics Belgium, Statistics Denmark, Statistics Netherlands, Statistics Sweden and Statistics Norway

Percentile	Belgium	Denmark	Netherlands	Sweden	Norway
	*k*200	*k*200	*k*200	*k*200	*k*200
10	100	100	100	0	100
25	100	100	100	0	141
50	141	141	100	250	200
75	224	224	141	250	412
90	424	1000	224	1414	1486
95	608	1513	500	2236	2500
99	1105	2200	1265	5000	6135

	*k*51,200	*k*51,200	*k*51,200	*k*51,200	*k*51,200
10	1500	1664	1712	2000	2561
25	2865	3354	2302	3162	4472
50	5049	7912	3612	10,050	11,005
75	7200	15,008	6379	22,472	29,221
90	9411	20,132	9080	35,609	56,292
95	12,394	23,308	10,515	44,294	73,645
99	20,096	36,111	14,091	104,346	139,384

### Measures of Segregation

#### Concentration

A concentration measure of segregation is obtained through Equipop, which calculates the proportion of non-European immigrants among the *k*-nearest neighbours of each grid cell. Weighted by the number of residents in each cell, the percentiles of the distribution of these neighbourhood compositions correspond to the percentile distribution of all individuals’ neighbourhood composition. The interpretation of the percentile values is straightforward; if, for instance, the 10th percentile is 1%, 10% of the population resides in neighbourhoods where 1% or less of the population are non-European immigrants.

#### Representation

Our measure of the representation of non-European immigrants is calculated from the percentile distribution of the concentration of non-European immigrants in a fashion identical to that in Andersson et al. (2018). Thus, non-Europeans are overrepresented in a percentile bin if the value is above 1, and under-represented if the value is below 1 (Andersson et al. 2017, 2018; Hennerdal and Nielsen 2017).

#### Dissimilarity Index

We calculate the DI for each *k* level based on the percentile distribution of our concentration measure, in the same fashion as Andersson et al. (2018). The DI is an aggregate measure of over- and under-representation that will be zero in the case of perfectly even representation, and one if the non-European population is perfectly segregated from the rest of the population.

## Results

The proportion of non-European immigrants in the different countries in 2011 and 2015 is summarized in Table 3. The lowest proportion can be found in Demark, followed by Norway, Belgium and the Netherlands, while it is the highest in Sweden. This order has remained stable, despite increasing proportions in all countries.

**Table 3:** Population share of non-European migrants in Belgium, Denmark, the Netherlands, Sweden and Norway, per cent. *Source*: Andersson et al. (2018), authors’ data and Eurostat

Country	2011 (%)	2015 (Jan., 1) born in non-member state (Eurostat) (%)
Denmark	4.8	6.9
Belgium	7.3	8.5
Netherlands	8.0	8.7
Sweden	9.1	11.1
Norway	5.7	7.8 (non-EU, foreign born)

### Concentration

**Fig. 1:**
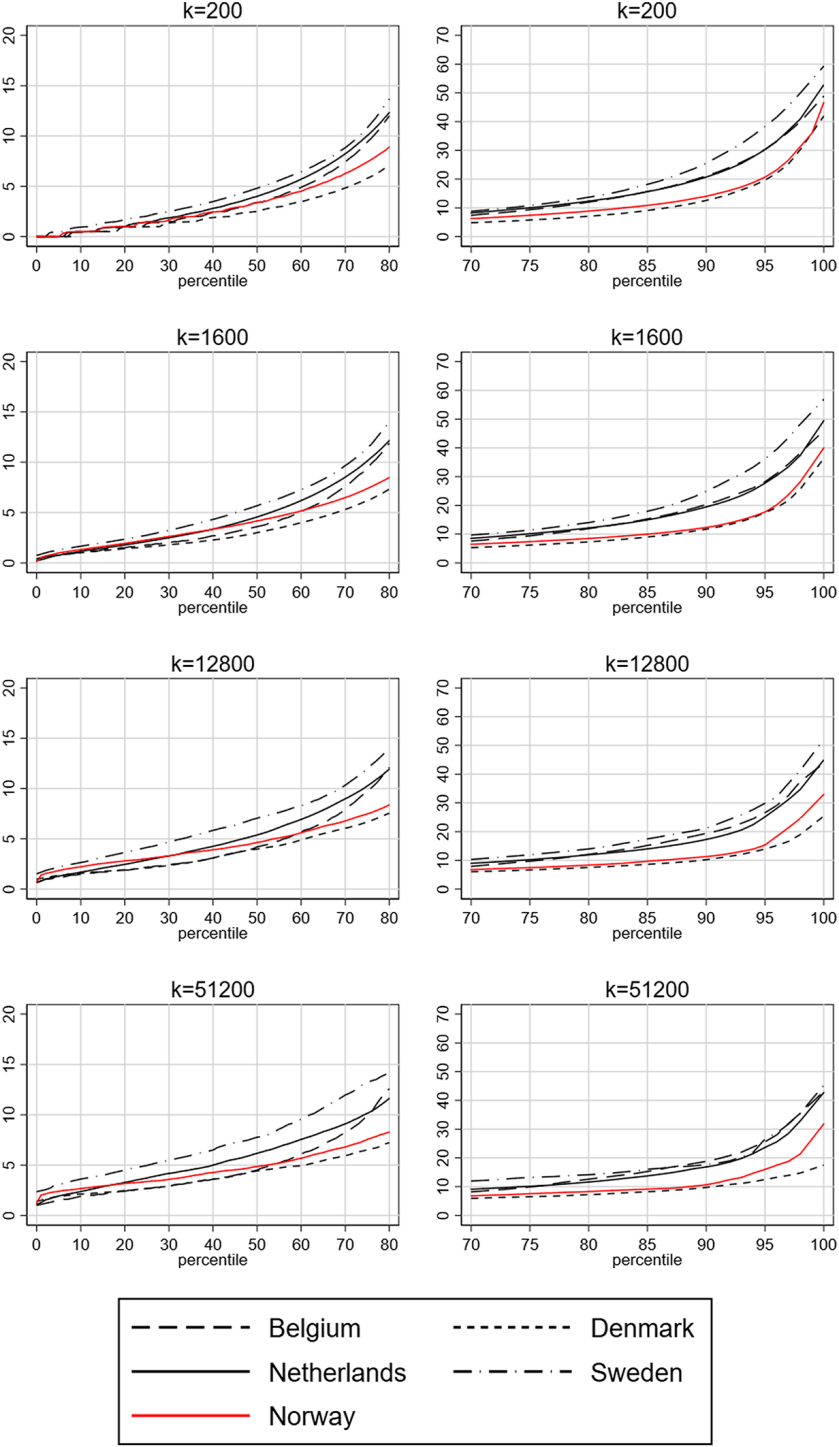
Concentration of non-European migrants in individualized neighbourhoods in Belgium, Denmark, the Netherlands, Sweden and Norway, 2011. Percentile values for *k* levels 200, 1600, 12,800, and 51,200. Lower percentiles in column one and percentiles above 70 in column two

The concentrations of non-European immigrants for each percentile and *k* level are plotted in Fig. 1. The left-hand column shows the concentrations in the lower part of the percentile distribution, while the right-hand column shows the higher part. The concentration of non-European immigrants closely follows the pattern found in the other countries up to about the 50th percentile, at all *k* levels. Above the 50th percentile, the neighbourhood concentrations in Norway closely resemble those in Denmark at the micro-level, but with slightly higher concentration levels. The concentration of non-European immigrants among the 200 nearest neighbours in Norway only exceeds 20% around the 95th percentile, telling us that 95 per cent of the Norwegian population lives in neighbourhoods where non-European immigrants constitute less than 20% of their 200 nearest neighbours. The exception to the resemblance with Denmark is at higher *k-*levels, where the Norwegian neighbourhoods with the highest concentration levels have much higher concentrations of non-European immigrants. This is indicative of macro-scale segregation patterns in Norway, likely related to ethnic segregation in and around the capital city Oslo, where the highest concentrations of non-European immigrants can be found. This result also illustrates the importance of considering segregation at different scales (Reardon et al. 2008). However, compared to Sweden, the Netherlands and Belgium, concentration levels in Norway are for the most part relatively modest at all neighbourhood scales. Selected percentile values are provided as supplementary material (S1).

### Representation

Our measure of representation also tells an interesting story about segregation in Norway; non-European immigrants appear more evenly represented in Norway than in the other countries—especially at low and intermediate neighbourhood scales of *k* = 200, *k* = 1600 and *k* = 12,800. As noted, a horizontal line at 1% would indicate perfectly even representation in all percentile bins. Although the differences are modest, the representation measure is overall closer to 1% in Norway than in the other countries at the low and intermediate scale levels. This suggests that non-European immigrants are more evenly distributed across the country in Norway.

**Fig. 2:**
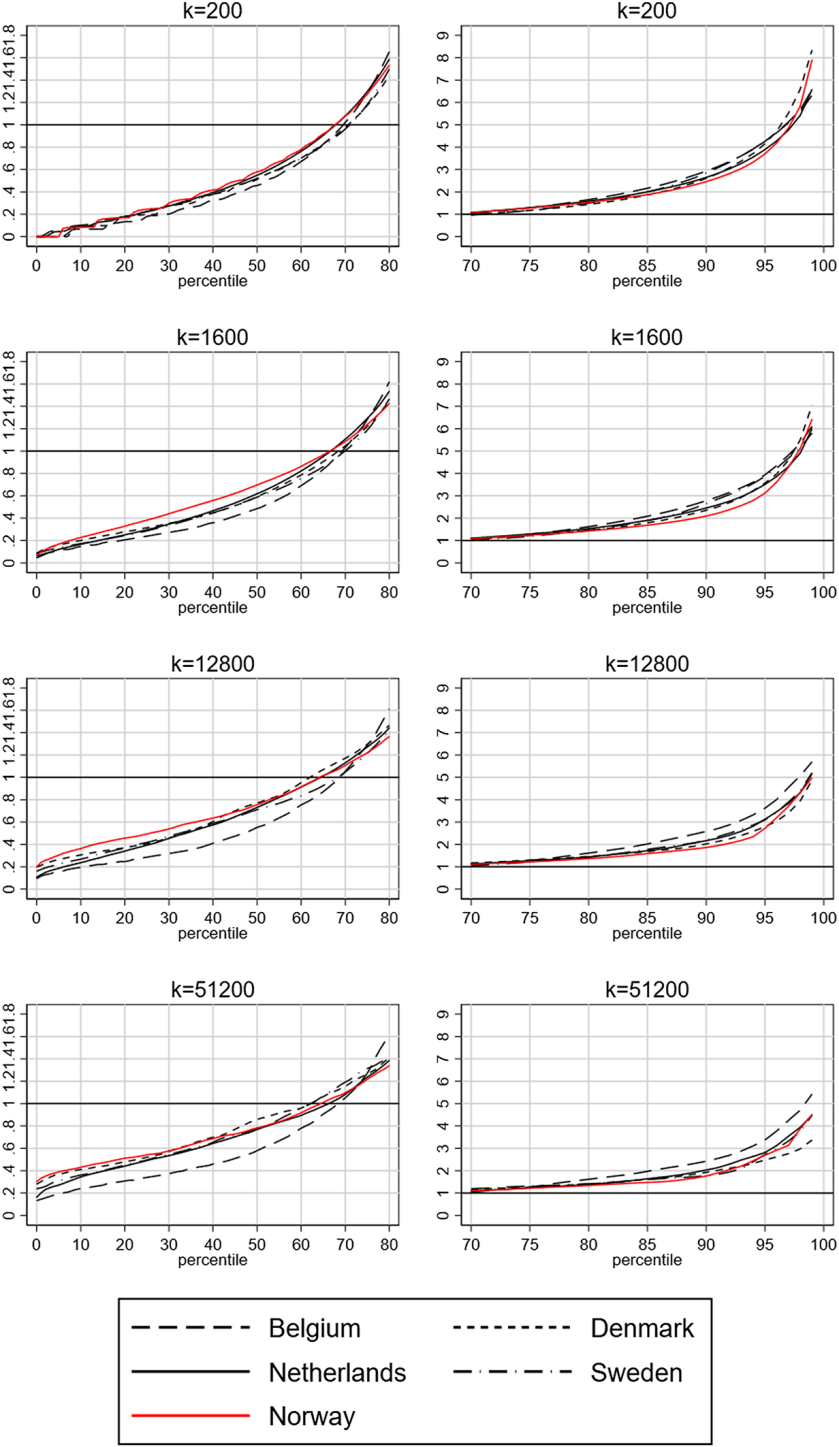
Representation of non-European migrants in 1% population bins, 2011. Population bins sorted according to the proportion of non-European migrants and diagrams showing different *k* values. Left column showing under-representation (below 1%, which is at the top of the diagram) and moderate and strong under-representation with 0.5% and 0.2%. Right column illustrating over-representation above 1% and moderate and strong over-representation at 2.0% and 5.0% non-European migrants in a bin. See online appendix to Andersson et al. (2018) for a discussion of these values

The DI for the neighbourhood bins (Table 4) largely confirms the impression from Fig. 2. Belgium has the highest DI at all levels, indicating stronger segregation. At the micro-level of *k* = 200, the DI values are of similar magnitude in Denmark, the Netherlands and Sweden, at between 47.5 and 48.9%, but lower in Norway (42.9%). At higher *k* levels, the values for Sweden and the Netherlands remain similar, while the levels in Norway and Denmark converge. The DI is lower in Norway than in Denmark at *k* = 1600 and *k* = 12,800, but slightly higher at *k* = 51,200.

**Table 4:** Dissimilarity index in Belgium, Denmark, Netherlands, Sweden and Norway, 2011. *Source*: Andersson et al. (2018), authors’ calculations based on register data from Statistics Belgium, Statistics Denmark, Statistics Netherlands, Statistics Sweden and Statistics Norway

*k* value	Belgium (%)	Denmark (%)	Netherlands (%)	Sweden (%)	Norway (%)
200	51.2	47.5	48.7	48.9	42.9
1600	47.3	40.4	43.6	44.1	35.9
12,800	43.7	31.3	37.5	35.7	29.2
51,200	40.6	25.3	32.6	29.7	26.2

Results based on all immigrants and non-western immigrants are provided as supplementary material (S2, S3). The pattern for all immigrants differs from those of non-European and non-western immigrants in a way that suggests that European immigrants are more evenly represented than other groups.

## Discussion and Conclusions

Andersson et al. (2018) found that small-scale segregation patterns of non-European migrants are similar across Belgium, Denmark, the Netherlands and Sweden, indicating a striking consistency in small-scale segregation levels across contexts. The segregation patterns presented here for Norway contradict this consistency and illustrate the diversity of segregation patterns, also at the small scale. We find overall segregation levels, as measured by the DI, to be lower in Norway at the neighbourhood scales of 200 and 1600 nearest neighbours than in the four countries studied by Andersson et al. (2018), and we find non-European immigrants to be more evenly represented across the country here than in the other national contexts. Concentration patterns in Norway are similar to those found in Denmark, but with higher concentration levels in the most immigrant-dense neighbourhoods at the macro-scale.

There are several candidate explanations for these patterns. One is the high prevalence of owner-occupied housing, which may contribute to a relatively even representation of non-European immigrants in Norway. Our findings are in line with those of Skifter Andersen et al. (2016) for Scandinavian capitals, which would support this idea. However, since we consider entire countries, and not just urban segregation, we believe that settlement policies for refugees and asylum seekers may be central to explaining why Norway displays a relatively even representation and low concentration levels. These policies may work in tandem with housing policies, policies aimed at maintaining the rural population, and a high rural employment rate, as well as universal social and welfare policies, making it relatively more attractive to remain in rural areas and small towns after initial settlement. Finally, a distinct pattern of macro-level segregation in Oslo between the east and the west (cf. Wessel 2017) may explain the higher concentration at the top of the distribution at high *k* levels in Norway compared to Denmark. Unfortunately, our data do not allow us to test these different explanations directly.

In sum, our results emphasize the highly variable and context-dependent nature of segregation patterns, the importance of comparable measures of segregation in comparative research, and the relevance of geographical scale in the study of segregation. Also, our results are consistent with the notion put forth by Andersson et al. (2018); non-European migrants are not only concentrated in migrant-dense areas. To the contrary, they are represented more evenly across the country in Norway than in Belgium, Denmark, the Netherlands and Sweden, especially at smaller geographical scales.

## Electronic supplementary material

Below is the link to the electronic supplementary material.

Supplementary material 1 (PDF 198 kb)

## Footnotes

1 Urban Europe, the Joint programming initiative (JPI), with partners in Belgium, Denmark, Norway, the Netherlands and Sweden, in the project “ResSegr—Residential segregation in five European countries. A comparative study using individualized scalable neighbourhoods”.
