# Supplementary material for: Neighbourhood Concentration and Representation of Non-European Migrants: New Results from Norway
Source: Eur J Popul. 2019 Mar 12;36(1):71–83. doi: 10.1007/s10680-019-09522-3 (PMC7018900; doi:10.1007/s10680-019-09522-3)
Supplement: Supplementary file 1 — Supplementary material 1 (PDF 198 kb) [file 10680_2019_9522_MOESM1_ESM.pdf]

SUPPLEMENTARY MATERIAL

S1. Concentration of non-European immigrants in Belgium, Denmark, Netherlands, Sweden and Norway, percentiles for different scales (k-levels), 2011.

Percentile	Belgium	Denmark	Netherlands	Sweden	Norway
	k = 200	k = 200	k = 200	k = 200	k = 200
10	0.5%	0.5%	0.5%	0.9%	0.5%
25	1.3%	1.0%	1.4%	2.0%	1.4%
50	3.4%	2.5%	4.0%	4.8%	3.4%
75	9.4%	5.8%	10.0%	10.8%	7.4%
90	21.1%	12.6%	20.7%	25.5%	14.0%
95	30.3%	19.6%	30.2%	38.3%	20.7%
99	44.4%	36.1%	46.8%	54.6%	38.8%
	k = 1600	k = 1600	k = 1600	k = 1600	k = 1600
10	1.1%	1.0%	1.2%	1.7%	1.3%
25	1.8%	1.6%	2.1%	2.8%	2.3%
50	3.6%	3.0%	4.6%	5.7%	4.2%
75	9.4%	6.2%	10.1%	11.4%	7.4%
90	20.3%	11.7%	19.5%	24.9%	12.3%
95	28.2%	17.5%	27.7%	36.2%	17.7%
99	42.1%	31.0%	43.4%	52.6%	34.1%
	k = 12,800	k = 12,800	k = 12,800	k = 12,800	k = 12,800
10	1.5%	1.6%	1.7%	2.6%	2.2%
25	2.2%	2.1%	2.9%	4.1%	3.0%
50	4.2%	4.0%	5.4%	7.1%	4.6%
75	9.7%	6.7%	10.2%	12.0%	7.5%
90	19.4%	10.3%	17.3%	21.2%	11.3%
95	26.6%	13.9%	25.1%	29.9%	15.3%
99	40.6%	22.2%	39.8%	46.7%	28.8%
	k = 51,200	k = 51,200	k = 51,200	k = 51,200	k = 51,200
10	1.9%	2.1%	2.4%	3.6%	2.7%
25	2.7%	2.7%	3.7%	5.0%	3.4%
50	4.5%	4.4%	6.2%	7.8%	4.9%
75	9.9%	6.5%	10.1%	13.2%	7.5%
90	18.8%	9.8%	16.8%	17.7%	10.6%
95	25.6%	12.5%	23.6%	26.5%	16.0%
99	39.3%	16.1%	37.8%	40.4%	26.6%

Source: Andersson et al. (2018), authors' calculations based on register data from Statistics Belgium, Statistics Denmark, Statistics Netherlands, Statistics Sweden and Statistics Norway.

S2. Concentration and representation for three different immigrant groups in Norway.

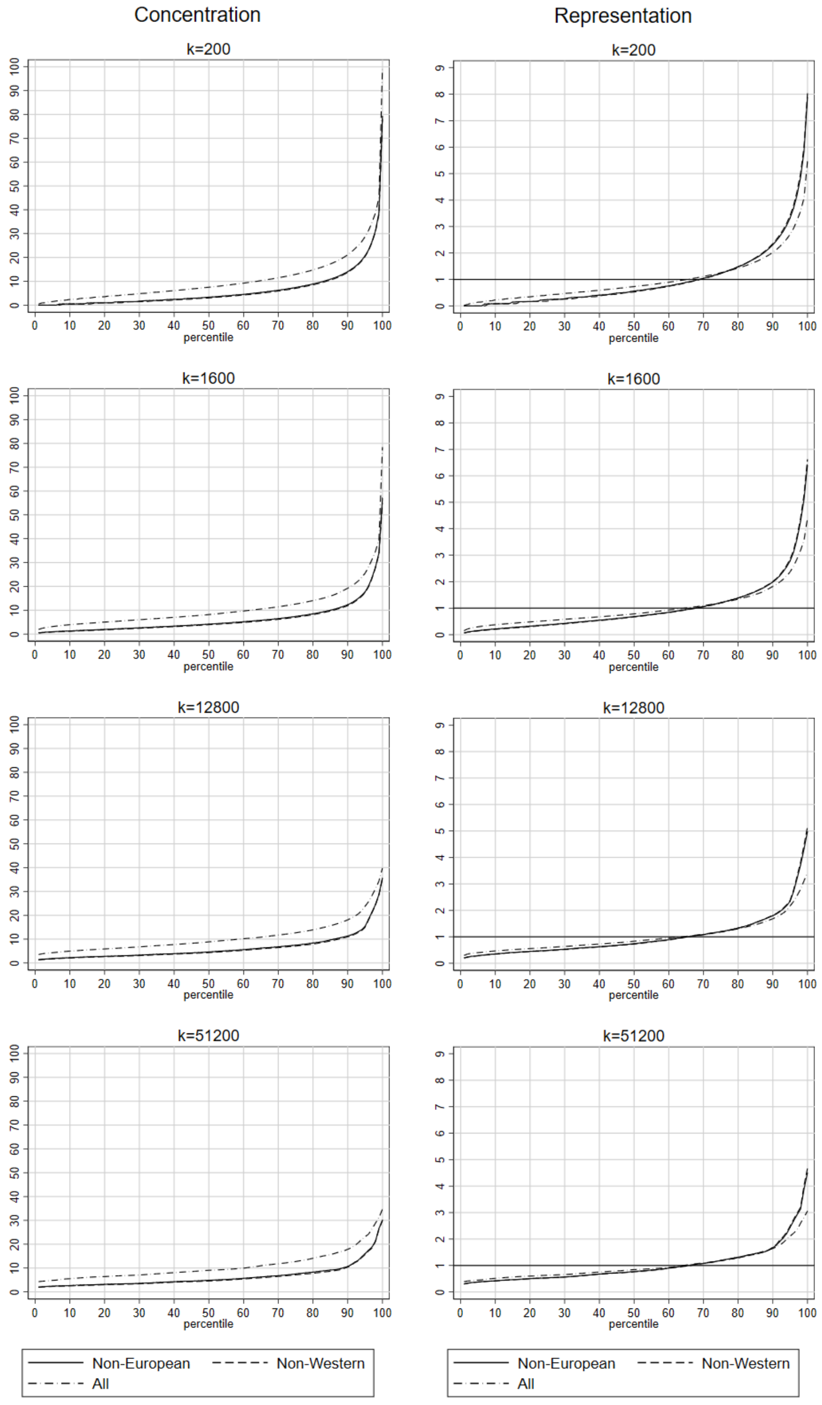

Note: Non-European immigrants comprise immigrants from all countries except counties except EU28/EFTA. Non-Western immigrants comprise immigrants from all counties except EU28/EFTA, North America, Australia and New Zealand. The horizontal line indicates even representation. Source: Authors' calculations based on register data from Statistics Norway

S3. Dissimilarity index for three different immigrant groups.

k-value	Non-European immigrants	Non-western immigrants	All immigrants
200	45.2%	44.1%	35.1%
1,600	37.5%	36.8%	28.2%
12,800	31.7%	29.7%	23.7%
51,200	27.4%	26.5%	22.0%

Source: Authors' calculations based on register data from Statistics Norway
